# Using the global value chain framework to analyse and tackle global environmental crises

**DOI:** 10.1007/s40812-022-00253-x

**Published:** 2023-01-21

**Authors:** Valentina De Marchi, Gary Gereffi

**Affiliations:** 1 Department of Economics and Management ‘Marco Fanno’, University of Padova, Via del Santo 33, 35123 Padua, Italy; 2 Emeritus Professor, Department of Sociology, Duke University, NC 27708 Durham, USA

**Keywords:** Climate crisis, Adaptive strategies, Global value chains, Upgrading, Governance, Sustainability, Q56, R11, F18

## Abstract

Climate crises are being experienced all over the world and appear to be accelerating as “extreme weather” events become the “new normal.” In today’s world economy, where trade and production activities are internationally dispersed and prone to disruptions, the global value chain (GVC) framework provides a systematic approach to understand and combat environmental crises and to advance sustainable development options across global, regional, and local scales. A vast “implementation deficit” characterizes sustainability efforts to date. The GVC framework incorporates firm and policymaker perspectives in a multistakeholder approach that offers multiple building blocks for a progressive environmental agenda, including: a multi-actor perspective to define sustainability; measuring it across diverse geographic scales; analysis of both environmental upgrading and downgrading; distinguishing motivations, actions, and outcomes when assessing environmental performance; viewing GVC resilience in terms of the interplay of economic and environmental forces; and highlighting how context matters in analyzing national, industry, and geopolitical factors.

## Introduction: environmental crises are global

Two years into the pandemic, the crisis generated by the spread of Covid-19 with its rapid and profound global impact appeared to be the apex of upheavals affecting our society and economy. While we are in an era of pervasive disruptions (Gereffi et al., 2022), in this piece we focus on the climate crisis. In particular, an intensification of heat waves and droughts, more frequent floods, wildfires and hurricanes, and a dramatic reduction in glaciers and rising sea-water levels suggest that the pandemic constitutes only one of a series of contemporary crises. Experts suggest that such climate crises are now experienced all over the world and are expected to exponentially increase in the immediate future (Intergovernmental Panel on Climate Change, 2022), so that ‘extreme’ events will increasingly become the ‘new normal’.

**Fig. 1:**
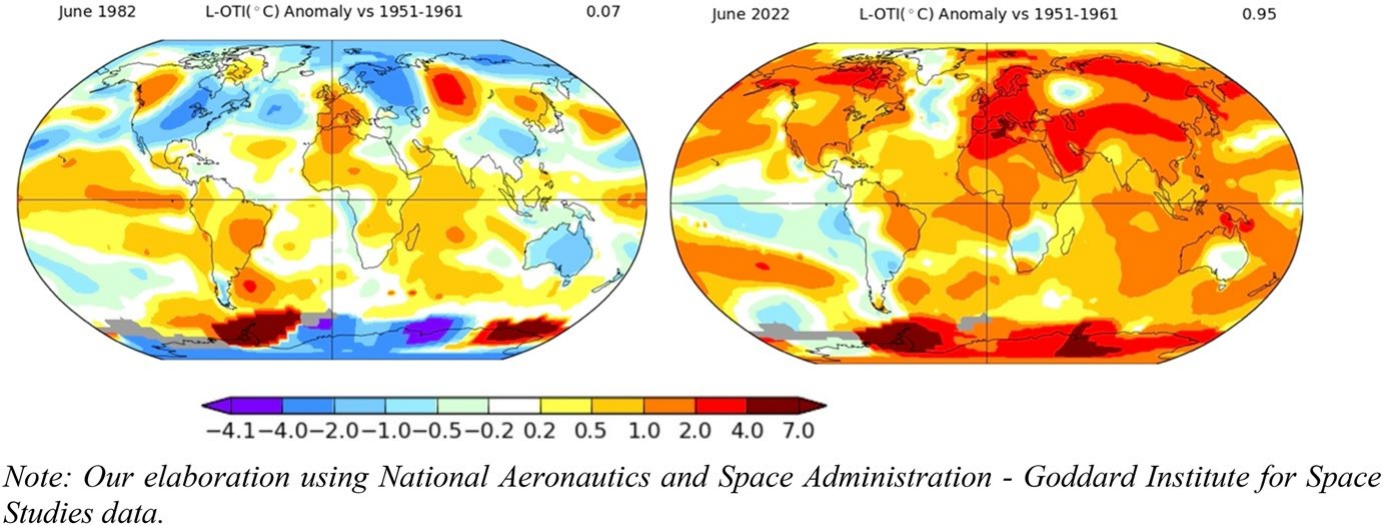
Temperature anomalies in June 1982 (left) and June 2022 (right), compared to 1951–1961

Clearly, climate crises have a global dimension. On the one hand, they are experienced throughout the world. Take changes in temperatures as an example—40 years ago they were experienced rather heterogeneously across regions, some being colder, others hotter than the reference period. However, in 2022 all regions show an intense increase in temperatures (+ 0.95 °C on average as respect to the decade 1951–1961) (see Fig. 1). Of course, the fact that the crisis is global does not mean that its manifestations are even across regions: on the contrary, it is expected that impacts of climate change are going to take different forms in every region (Intergovernmental Panel on Climate Change, 2022). In the same year, it is likely that some regions will experience higher desertification and droughts, while others will face increasing floods and abnormally heavy precipitation.

Looking at Italy and Pakistan in 2022 provides a clear example of this duality: Italy confronted a terrible drought, with rainfall levels 45% below the usual average; meanwhile, in Pakistan a torrential monsoon unleashed one of the most devastating floods in the country’s history and one of the world’s deadliest. Despite opposite outcomes, the underlying cause is the same—climate change—which is just one of the many crises we will be facing (Steffen et al., 2015).

On the other hand, the human activities motivating those crises also have a global footprint (Intergovernmental Panel on Climate Change, 2022). Production and consumption activities, indeed, are responsible for a large share of emissions. According to Climate Watch data, as of 2016, among the 49.4 tonnes of CO_2_ equivalent worldwide, 29.4% are emerging from energy use in industry—including emissions related to transport of goods and people (16.2%), and emissions related to agriculture, forestry and land use (18.4%). Indeed, to provide a thorough account of the carbon footprint of firms, it is important to consider not only their direct emissions (Scope 1), but also the emissions generated from the purchase of electricity, steam, heating and cooling for running the company’s activities (Scope 2)^1^ and even the emissions from suppliers that generate the inputs and components needed by the firm to perform their activities (Scope 3).

**Fig. 2:**
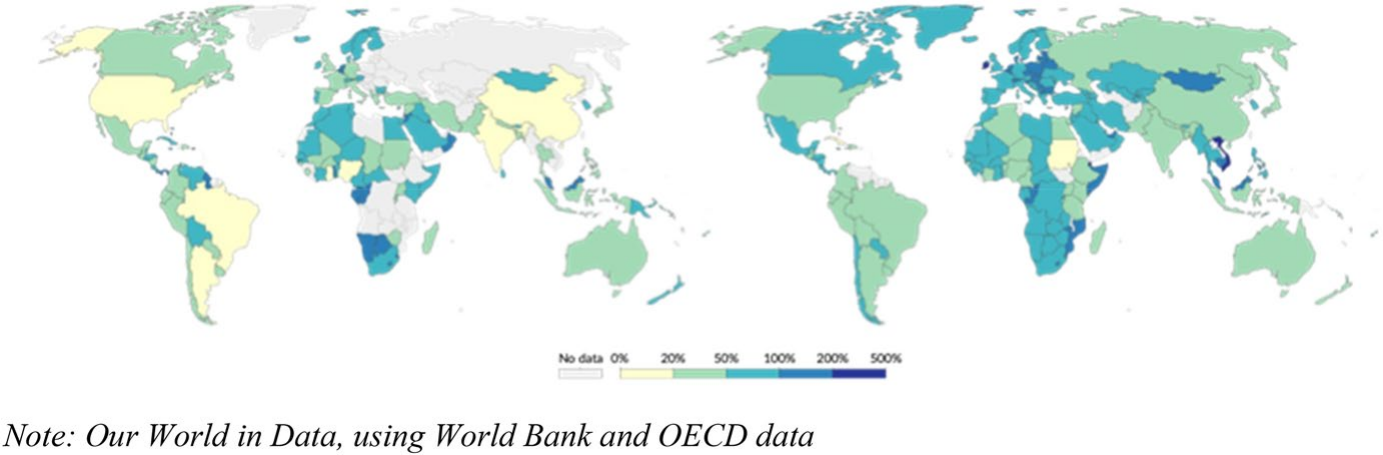
Export and imports of goods and services as share of GDP in 1982 (left) and 2020 (right)

These production and consumption activities are indeed global. Figure 2 proxies the increasing globalization of production by reporting exports and imports as a share of gross domestic product (GDP) worldwide, comparing 1982 and 2020. The world economy clearly became more interconnected through trade over the past four decades, although the recent COVID-19 pandemic has raised significant questions about future trends (see the articles in the special issue edited by Panwar et al., 2022).

As manufacturing is outsourced across different countries to pursue diverse location advantages, production activities are increasingly globally dispersed. Because the causes and consequences of climate crises are global by nature, a global scale solution is also required and for that we need a systems perspective. The actions of single actors—businesses, non-governmental organizations (NGOs), the state, and other institutions—must be framed in an integrated and systemic way to address environmental grand challenges and identify solutions (Bansal & Song, 2017; Grewatsch et al., 2021). Given the inherent complexities and tensions of today’s contemporary economy (Carmine & De Marchi, 2022; Waddock et al., 2015), the global value chain (GVC) framework can be a powerful tool that adopts a systems lens in understanding and forging responses to climate crises.

## Charting environmental sustainability along GVCs

Environmental sustainability is increasingly central in *scholarly* discussions of GVCs and the related international business (IB) domain (De Marchi et al., 2020; Golgeci et al., 2021; Lund-Thomsen & Lindgreen, 2014). Much of this literature has focused on the impact of governance mechanisms of global “lead firms” in driving a cascade of sustainability practices along the GVC (Alexander, 2020; De Marchi et al., 2013; Lund-Thomsen & Lindgreen, 2014). Within this realm, attention has been given to the role of (global and local) standards and codes of conduct, often developed within multistakeholder initiatives, and the conditions under which they might effectively drive change (Fransen & Kolk, 2007; Langford et al., 2022; Ponte & Gibbon, 2005). While most of this literature looks at the business practices implemented to reduce environmental impacts, there is a growing focus on the environmental outcomes accruing from those practices, viewed from the standpoint of the multiple actors spread across GVCs (Clarke & Boersma, 2015; Krauss & Krishnan, 2016; Krishnan et al., 2022).

The GVC dimension of the environmental agenda is also central for *practitioners’* main concerns, driven by increasing evidence of the scale of environmental impacts along global supply chains. Consider CO_2_ emissions as an example. It is estimated that supply chain emissions—the so-called Scope 3 emissions—are on average 11.4 times higher than operational emissions, particularly when considering product categories such as electronics, automotive, food or fashion (Carbon Disclosure Project, 2020; World Economic Forum & Boston Consulting Group, 2021).

A GVC perspective on environmental problems is being adopted by *policymakers* too, as they urge firms to be responsible for the environmental impacts of both downstream stages (e.g., the so-called Extended Producer Responsibility laws, which make producers responsible for post-consumption waste for certain product groups) and upstream stages (e.g., the due diligence rules introduced in the case of commodities associated with deforestation and forest degradation).^2^

## GVCs as a lens to examine environmental sustainability complexities

Despite environmental sustainability and green supply chain management becoming mainstream concerns in the operations of companies worldwide, the pace of improvements is lagging the urgency linked to escalating climate change disruptions. According to Accenture’s, 2022 survey of Global CEOs, while 72% agree that sustainability remains an immediate priority for their business and 49% are grappling with supply chain interruptions due to extreme weather events, half of the companies report their analysis of supply chain risks to be at either a basic level or non-existent. In addition, 48% recognize that extending their sustainability strategy across the entirety of their GVC is a top barrier to effective implementation (Accenture, 2022).

Thus, a vast “implementation deficit” characterizes sustainability efforts to date. Implementing sustainability is a complex issue that is simultaneously multifaceted (needing to account for different environmental aspects, such as biodiversity and acidification of soil), subjective (environmental problems are experienced differently by diverse actors, depending on their location and activity profile), and changing over time (what we considered sustainable 10 years ago is no longer sufficient today).

Against this backdrop, the GVC framework (Fernandez-Stark & Gereffi, 2019; Ponte et al., 2019) is uniquely positioned to better and more clearly understand how to tackle sustainability complexities throughout GVCs (Campling & Havice, 2019; De Marchi et al., 2019; Lund‐Thomsen, 2019). It adopts a broader view than the firm-level perspective typical of many IB studies, and it is more fine-grained than international economics approaches, which often use country-level data attuned to the interests of national policymakers (Gereffi, 2019). Several critical features of the GVC framework allow it to address the implementation deficit more effectively through an actor-centered approach that explicitly depicts how sustainability issues are experienced across a broad range of actors in GVCs, and the range of strategic options to address them.

First, it maps *how activities are sliced and geographically distributed across key actors*, enabling a clearer understanding of which activities and environmental impacts are linked to a specific node of production, and the major environmental pressures within and across particular locations and activities (Krishnan et al., 2022). Take for example the fashion apparel industry, which is the second most polluting industry after oil and gas. And in particular, take the example of leather products engaging numerous actors that transform skins into leather, and leather into accessories or clothing. How many of those input activities should be greened to ensure the final products can be considered green? Such analysis can help, for example, to determine whether deforestation should (or should not) be considered a key environmental issue attributable to the production of leather accessories since cattle skins in the Amazon are used in leather production worldwide (Mammadova et al., 2020; De Marchi and Di Maria 2019).

Second, by mapping *who is performing different value-added activities* and *how such activities are coordinated and governed*, GVC analysis helps to identify bottlenecks and the potential cascading of sustainability initiatives along GVCs. It can, for example, shed important light on which firms support the development and implementation of higher standards along the GVC, where value is extracted and accumulated, and which strategies can unite actors to counter environmental issues and/or the power of opposing actors (Havice & Campling, 2017).

Third, by analyzing *the role of the state and international institutions* in GVCs, GVC analysis can identify which forms of private and public governance can effectively drive environmental improvements. Private standards, even the most strict, are not implemented in a vacuum; if the country where they ‘touch down’ has a coherent regulatory framework and active civil society, codes of conduct are more likely to effectively achieve their intended goals (De Marchi & Alford, 2022; Gereffi & Lee, 2016).

## Building blocks for a progressive agenda of environmental upgrading in GVCs

The literature on environmental sustainability in GVCs is still nascent, relative to the more consolidated literatures on the economic and social dimensions of global production (Khattak & Pinto, 2018; for recent reviews, see De Marchi et al., 2020; Golgeci et al., 2021). To advance this field, we outline a GVC research agenda that provides guidelines for scholars seeking to understand how firms and related institutions can foster environmental sustainability in GVCs.

- *Adopt a multi-actor perspective to define sustainability.* Environmental sustainability is a contested concept; suppliers, lead firms, states and NGOs may adopt very different views about what sustainability is and should be (Krauss & Krishnan, 2016; Mammadova et al., 2020; Zimmermann et al., 2022). Thus, a first crucial step for academics is to adopt a multi-actor perspective that acknowledges that (un)sustainability will be experienced differently along the GVC and accounts for consensus and contradictions in actors’ perspectives. While a certain intervention might lead some actors to report improvements in their own environmental performance (e.g., lead firms committing to supply sustainably-certified raw materials), it may come at the cost of declining sustainability experienced by local actors (e.g., due to the increasing acidification of the soil driven by the type of agriculture required to achieve the certification scheme) (Krishnan et al., 2022). Research aiming at identifying the best practices to manage GVCs to tackle environmental problems should thus evaluate practices using a system perspective, measuring the ability to reduce overall impacts.
- *Measure sustainability as a multifaceted concept*. A key challenge in fostering environmental sustainability along GVCs is the difficulty in measuring it as a multifaceted concept. While measures of certain impacts are well established (e.g., CO_2_ emissions), for other environmental issues (such as biodiversity) widely accepted, universally valid measures do not exist. Furthermore, some elements of environmental sustainability are simply difficult to assess (e.g., how to determine the extinction of an animal species). This is compounded by a lack of systematic data collection by geographically dispersed GVC actors, particularly those operating in the lower tiers of the value chain, such as small and informal firms. Take the case of an electronic product: what would make it sustainable? Working to substitute traditional materials with more ecological ones (avoiding the use of ‘conflict minerals’, using recyclable or recycled materials) or is it rather to reduce the amount of materials used per unit of output, the long-term design and easy to repair?
- *Incorporate robust measures of both upgrading or downgrading.* Research aiming at identifying which practices can support the greening of GVCs should avoid the use of fuzzy measures of sustainability and rather adopt robust indicators of environmental improvements, avoiding the temptation to conflate this diversity into a one-size-fits-all measure. Thus, scholars should utilize interdisciplinary approaches and adopt multiple methods, drawing upon the most advanced technical methods to measure sustainability, while not renouncing qualitative methods associated with GVC analysis to provide depth and face validity in the description of difficult-to-measure elements. In this effort, researchers should be open to measure downgrading as well (often overlooked in relation to upgrading) and should avoid conflating up- and downgrading, as they may not represent opposite poles along the same continuum. Indeed, especially when it comes to firms that are active in different international locations, it is highly likely that they are both scoring important corporate social responsibility initiatives and at the same time, but—maybe in different subsidiaries—been also engaging in corporate misconducts, unethical procedures, infringements of human rights (Fiaschi et al., 2020; Nieri & Giuliani, 2018).
- *Distinguish between motivations, actions and outcomes of sustainability performance in GVCs.* When seeking to measure sustainability performance in GVCs, researchers should not fall in the trap of confusing motivations, actions and outcomes (Krishnan et al., 2022). Indeed, implementing sustainability practices along the GVC is no easy task—even highly committed firms might fail to reach their intended sustainability goals. Similarly, lead firms might promote several sustainability efforts (e.g., investing in changing production processes or in ensuring all raw materials are certified), yet fail to effectively address environmental problems by not targeting the most pressing problem (Halme et al., 2020). Thus, researchers should collect and analyze data not only on firm actions and motivations, but also on sustainability outcomes.
- *Resilience is fostered by combining environmental and economic upgrading.* It is not possible to discuss environmental upgrading opportunities without considering how actors capture the economic value created in the process (Ponte, 2019). Economic opportunities associated with the introduction of more environmentally friendly practices along GVCs (e.g., moving toward more eco-efficient product lines) shape how environmental upgrading is going to take place. Furthermore, environmental upgrading might be related to more resiliency in GVCs—a particularly relevant policy issue after the COVID-19 pandemic (Gereffi et al., 2022)—because sustainability-oriented supply chains require longer and more trust-oriented commitments, which also favor resiliency. Failure to account for the economic side of environmental upgrading might undermine the ability to understand its current and future development. This element is particularly relevant in contexts where populism is on the rise, as the trade-off between environmental and economic factors might reduce institutional support for a sustainable transition and divert investments to short-term programs with clearer economic incentives.
- *Context matters.* A key strength of the GVC framework is to facilitate analysis of the complex interconnections between ‘vertical’ inter-firm connections, and ‘horizontal’ institutional and geographical contexts. Adopting a GVC-focused approach allows for deeper inquiry into specific contextual factors in determining upgrading trajectories—e.g., industry specialization, presence of institutional actors, the role of the state, and local innovation capabilities (Gereffi et al., 2021; Pietrobelli & Rabellotti, 2011; Pietrobelli et al., 2021). Take the cases of the fashion industry and the maritime industry. In the former, greening has been driven mostly by external actors—NGOs are particularly active in this very polluting industry—who effectively drive the awareness of both firms and consumers. In the maritime industry, change has been driven mostly by internal-to-the-firm motivations (i.e. cost reduction), while no external drivers, not even regulation, proved effective to drive upgrading (De Marchi et al., 2019).

## Conclusion

We are in the countdown to reverse humanity’s path toward destroying essential planetary boundaries. In the fight to address climate crises, firms play a central role that is very often overlooked. The ‘economy’ is perceived as a monolithic actor, and key lead firms should be punished or nudged by policy makers, consumers, and NGOs. But the details of how firms might effectively reduce their overall environmental impacts, and the possibility for firm-firm interactions to drive better conditions in the challenges they confront, are not considered at the policy-making level or even within the social activism realm. Take the last book edited by Greta Thunberg (2022): among its many chapters, none are devoted to unpacking the ‘economy’ from a firm or GVC perspective. If production activities indeed are directly and indirectly responsible for a large amount of the environmental problems we are experiencing, then we must gain and act upon a deep understanding of the mechanisms that drive their activities. If we want to change global industries, we need to understand how they work.

The sustainability agenda is a top priority that requires novel insights and ambitious agendas from the scientific, business and policy-making communities. The COP27 Climate Summit that recently concluded in Sharm el Sheikh, Egypt is the most recent effort by world leaders to advance climate negotiations to cut greenhouse gas emissions amid a global energy crisis, war in Europe, and rising inflation. While there are deep tensions between rich polluting countries and poor nations bearing the brunt of climate impacts over who should pay the costs of global warming, the GVC framework offers a systematic approach in which both shared responsibilities and equitable policy options can be identified and debated, and research agendas can explore the most promising opportunities for sustainable development outcomes in key industries and locations.

## Data Availability

No data have been used to write this perspective article.

## Footnotes

1 Energy makes up 77% of EU green-house gas emissions.

2 See the ‘Proposal for a regulation of the European Parliament and of the council on the making available on the EU market as well as export from the EU of certain commodities and products associated with deforestation and forest degradation and repealing regulation (EU) no 995/2010’. https://data.consilium.europa.eu/doc/document/ST-14151-2021-INIT/en/pdf
